# Ion-Induced Synthesis of Alginate Fibroid Hydrogel for Heavy Metal Ions Removal

**DOI:** 10.3389/fchem.2019.00905

**Published:** 2020-01-10

**Authors:** Chuncai Kong, Xueqi Zhao, Yingju Li, Sen Yang, Yong Mei Chen, Zhimao Yang

**Affiliations:** ^1^School of Science, MOE Key Laboratory for Non-Equilibrium Synthesis and Modulation of Condensed Matter, Xi'an Jiaotong University, Xi'an, China; ^2^College of Bioresources Chemical and Materials Engineering, Shaanxi University of Science & Technology, Key Laboratory of Leather Cleaner Production, China National Light Industry, Xi'an, China

**Keywords:** fibroid hydrogel, sodium alginate, heavy metal ions removal, adsorption kinetics, adsorption isotherms

## Abstract

Design and synthesis of environmentally friendly adsorbents with high adsorption capacities are urgently needed to control pollution of water resources. In this work, a calcium ion-induced approach was used to synthesize sodium alginate fibroid hydrogel (AFH). The as-prepared AFH has certain mechanical strength, and the mechanical strength is enhanced especially after the adsorption of heavy metal ions, which is very convenient for the recovery. AFH exhibited excellent adsorption performances for Cu^2+^, Cd^2+^, and Pb^2+^ ions and displayed very high saturated adsorption capacities (Qe) of 315.92 mg·g^−1^ (Cu^2+^), 232.35 mg·g^−1^ (Cd^2+^), and 465.22 mg·g^−1^ (Pb^2+^) with optimized pH values (3.0–4.0) and temperature (303 K). The study of isotherms and kinetics indicated that adsorption processes of heavy metal ions fitted well with the pseudo-second-order kinetics model and the Langmuir model. Pb^2+^ was found to have the strongest competitiveness among the three heavy metal ions. Thus, AFH has great application prospects in the field of heavy metal ions removing from wastewater.

## Introduction

With rapid global development, water contamination caused by industrialization and a growing population has been an urgent problem that must be solved. Toxic heavy metal ions in wastes derived from the emission load of industry are the most harmful contaminants to human beings (Chen et al., 2009). Heavy metal elements are generally highly toxic, refractory, and tend to accumulate over time, causing great harm to human health. For instance, trace amounts of cadmium (Cd^2+^) in the human body eventually accumulate and causes various diseases related to the lungs, bone, kidneys, liver, immune system, and reproductive organs (McDowell, 2003). Because of its importance in enzyme synthesis, bone and tissue development Copper (Cu) is an indispensable trace element in the human body. However, divalent copper (Cu^2+^) ion is carcinogenic and toxic, resulting in a variety of complications and liver cirrhosis once it accumulates in the viscera (Bilal et al., 2013). Lead (Pb^2+^) is non-biodegradable and tends to accumulate in living tissues, leading to various kinds of disorders and diseases (Chen et al., 2010). Thus, it is urgent to solve the problem of heavy metal ions contamination of water.

To date, four common methods (including physical, chemical, physiochemical, and biological) have been developed to remove heavy metal ions from wastewater (Wang et al., 2003; Kurniawan et al., 2006; Wang and Chen, 2009; Fu and Wang, 2011; Chukwuemeka-Okorie et al., 2018). Physical methods include evaporation and dilution. Chemical methods mainly contain chemical precipitation method and electrolysis method. Biological methods are those in which microorganisms are used for degradation. Physicochemical methods are processes like adsorption, ion reduction, and ion exchange. Adsorption has the advantages of low cost, easy to obtain, availability, and environmentally friendly, thus, it has been widely applied in industrial effluent treatments. Hence, many kinds of adsorbents have been designed and synthesized (including silica, magnetic particles, grapheme, and hydrogel) to meet the demands for water treatment (Liu et al., 2013; Wang et al., 2015; Hayati et al., 2017; Zhao et al., 2017; Alizadehgiashi et al., 2018; Qi et al., 2019).

Hydrogel is one of the excellent adsorbents that has three-dimensional (3D) hydrophilic polymer networks and is capable of absorbing ions and retaining water within their networks. Removal of heavy metal ions using hydrogel involves the adsorption through interactions with functional groups dangling on polymer chains such as carboxyl, hydroxyl and amino groups (Kaşgöz et al., 2003; Zhou et al., 2018). Luo reported a tough polyampholyte/graphene oxide hydrogel adsorbent for the removal of Pb^2+^ (216.1 mg·g^−1^) and Cd^2+^ (153.8 mg·g^−1^) ions (Zhou et al., 2016). Wang reported a freeze-thaw method to synthesize PVA/CMC hydrogels with a high degree of crosslinking, and the hydrogels showed excellent adsorption capacities for heavy metal ions including Ag^+^ (8.4 mg·g^−1^), Cu^2+^ (5.5 mg·g^−1^), Ni^2+^ (6.0 mg·g^−1^), and Zn^2+^ (5.3 mg·g^−1^) (Wang and Wang, 2016). Though lots of efforts have been made to prepare various hydrogel-based composites for the removing of heavy metal ions, facile design, and synthesis of environment friendly hydrogels with high adsorption capacities are still urgently needed.

For this purpose, we developed a calcium ion (Ca^2+^)-induced crosslinking approach to fabricate sodium alginate fibroid hydrogel (AFH). AFH was used for the removal of Cd^2+^, Cu^2+^, and Pb^2+^ ions and the effects of temperature, adsorption time, pH values, and interfering ions on the adsorption capacity and removal ratio were systematically investigated. The optimized conditions of AFH for adsorption behaviors were analyzed via adsorption kinetics and adsorption isotherms. AFH was capable of efficiently removing heavy metal ions and had very high saturated adsorption capacities (*Q*_*e*_) for Cu^2+^ (315.92 mg·g^−1^), Cd^2+^ (232.35 mg·g^−1^), and Pb^2+^ (465.22 mg·g^−1^) ions. The easily prepared AFH with high adsorption performances for various heavy metal ions has great potential in the controlling pollution of water resources.

## Materials and Methods

### Materials

Sodium hydroxide (NaOH), calcium sulfate (CaSO_4_), lead nitrate [Pb(NO_3_)_2_], cupric nitrate [Cu(NO_3_)_2_•3H_2_O], calcium nitrate [Cd(NO_3_)_2_•4H_2_O], Sodium alginate (SA), and hydrochloric acid (HCl) were obtained from Aladdin Reagent. All these chemicals were used without further purification.

### Methods

#### Preparation of Alginate Fibroid Hydrogel

In a typical synthesis of AFH, 5.0 g of SA was added to 95.0 mL of deionized water at room temperature and stirred for 5 h to produce a polymer solution. A 10 mL injection syringe (Φ16 mm) was then used to inject the as-prepared dispersion of SA into a super-saturated solution of CaSO_4_. After 30 min of crosslinking, the product was rinsed three times with deionized water to form the desired hydrogel. The prepared hydrogels were dried at 40°C for 24 h before the adsorption of heavy metal ions.

#### Preparation of Heavy Metal Ions Stock Solutions

Stock solutions of each heavy metal ions of 1,000 mg•L^−1^ were prepared. Exact amounts of 2.744 g of Cd(NO_3_)_2_•4H_2_O, 3.802 g of Cu(NO_3_)_2_•3H_2_O, and 1.599 g of Pb(NO_3_)_2_ were each dissolved in 1,000 mL of Milli-Q water, respectively. Solutions with certain required concentrations could be obtained by diluting the as-prepared stock solution.

#### Characterizations

Morphologies of the as-prepared materials were investigated using a field-emission scanning electron microscope JSM-7000F (FE-SEM, JEOL, Japan). Thermal gravimetric analysis (TGA) of the production was carried out on an SDT-Q600 (TA instruments, USA) under Ar atmosphere from 25°C to 800°C with a heating rate of 10°C•min^−1^. ICP-OES was carried out on Optima 8000 (PerkinElmer, USA). A tensile tester (SHIMADZU AGS-X) with a 500 N load cell was used for the tensile test. The cylindrical hydrogel samples with or without heavy metal ions adsorbed were selected for all tests. The upper clamp was pulled by the load cell at a constant velocity of 50 mm/min.

#### Adsorption of Heavy Metal Ions

Adsorption of metal ions by AFH were carried out as follows: A certain quantity of as-prepared dried hydrogels was added into the heavy metal aqueous solution with a certain concentration. After a specified time of adsorption reaction, aliquots of the solutions were collected to test the adsorbed heavy metal ions concentration using ICP-OES.

The equilibrium adsorption capacity (*Q*_*e*_, mg·g^−1^) is defined as the amount of heavy metal adsorbed per gram of dried gel at equilibrium. *Q*_*e*_ can be obtained by Equation (1) (Zhang et al., 2018):

(1) $$\begin{array}{l} Q_{e}=\frac{\left(C_{0}-C_{e}\right)V}{M} \end{array}$$

where *C*_0_ and *C*_*e*_ (mg·L^−1^) are the initial and equilibrium concentrations, respectively, for each metal ion in solution; *V* (L) is the solution volume and *M* (g) is the mass of the dried hydrogel.

The removal ratio (*R*) for each metal ion in solution at a specific time can be calculated by Equation (2)

(2) $$\begin{array}{l} R=\frac{\left(C_{0}-C_{t}\right)}{C_{0}}\times 100\% \end{array}$$

where *C*_*t*_(mg·L^−1^) is the concentration of each metal ion at a specific time.

### Adsorption Kinetics

The main rate-limiting factor can be determined through adsorption kinetics. Equations (3) and (4) represent the first-order kinetics and second-order kinetics, respectively (Li et al., 2016).

(3) $$\begin{array}{l} \mathrm{lg}\left(Q_{e}-Q_{t}\right)=\mathrm{lg}Q_{e}-\frac{K_{1}t}{2.303} \end{array}$$

(4) $$\begin{array}{l} \frac{t}{Q_{t}}=\frac{1}{K_{2}Q_{e}^{2}}+\frac{t}{Q_{e}} \end{array}$$

where *Q*_*e*_ (mg·g^−1^) and *Q*_*t*_ (mg·g^−1^) are the equilibrium adsorption capacities and the adsorption capacities at specific time *t* (h), respectively. *t* (h) is the reaction time, *K*_1_ (h^−1^) is the first-order kinetics rate constant, and *K*_2_ (g·(mg·min)^−1^) is the second-order kinetics rate constant.

#### Adsorption Isotherms

The adsorption behaviors of AFH can by described by the Langmuir and Freundlich isotherm models (Zhang et al., 2017). The Langmuir isotherm model can be represented by Equations (5) and (6):

(5) $$\begin{array}{l} Q_{e}=\frac{Q_{m}K_{L}C_{e}}{1+K_{L}C_{e}} \end{array}$$

(6) $$\begin{array}{l} \frac{C_{e}}{Q_{e}}=\frac{C_{e}}{Q_{m}}+\frac{1}{K_{L}Q_{m}} \end{array}$$

where, *C*_*e*_ (mg·L^−1^) is the equilibrium concentration for each metal ion, *Q*_*e*_ (mg·g^−1^) is the equilibrium adsorption capacity, *Q*_*m*_ (mg·g^−1^) is the theoretical saturation adsorption capacity and *K*_*L*_ is the Langmuir isotherm model constant.

In the Langmuir isotherm adsorption process, the separation factor (R_L_) can be used to evaluate the pros and cons of an adsorption system (linear, irreversible, favorable and unfavorable) (Meena et al., 2005). *R*_*L*_ was calculated according to Equation (7):

(7) $$\begin{array}{l} R_{L}=\frac{1}{1+K_{L}C_{0}} \end{array}$$

Freundlich isotherm model can be written by Equations (8) and (9):

(8) $$\begin{array}{l} Q_{e}=K_{F}C_{e}^{\frac{1}{n}} \end{array}$$

(9) $$\begin{array}{l} \log Q_{e}=\log K_{F}+\frac{1}{n}\log C_{e} \end{array}$$

where *C*_*e*_ (mg·L^−1^) is the equilibrium concentration for each metal ion, *Q*_*e*_ (mg·g^−1^) is the equilibrium adsorption capacity, *K*_*F*_ is the Freundlich isotherm constant and *n* is the Freundlich isotherm constant and the adsorption process is favorable when the value of *n* is between 1 and 10.

#### Competitive Adsorption

The adsorption competitiveness of different metal ions can be written as Equations (10) and (11) (Yan et al., 2011):

(10) $$\begin{array}{l} K_{d}=\frac{\frac{Q_{i}}{C_{i}}}{\frac{Q_{1}}{C_{1}}+\frac{Q_{2}}{C_{2}}+\cdots \frac{Q_{j}}{C_{j}}} \end{array}$$

(11) $$\begin{array}{l} \;\alpha =\frac{K_{d}\left(T\right)}{K_{d}\left(I\right)} \end{array}$$

where *K*_*d*_ is the partition coefficient of a certain metal ion, *i* = 1, 2.. *j*, and *j* = 1, 2,…, *Q*_*i*_ (mg·g^−1^) and *C*_*i*_ (mg·g^−1^) are the equilibrium adsorption capacity and initial concentration of a certain metal ion. α is the selectivity coefficient of a certain metal ion. *K*_*d*_*(T)* is the partition coefficient of ions with stronger competitiveness, and *K*_*d*_*(I)* is the partition coefficient of ions with weaker competitiveness. In the adsorption system, the sum of the partition coefficient values is 1, and the larger the value, the stronger the competitiveness of the ions.

## Results and Discussion

**Figure 1:**
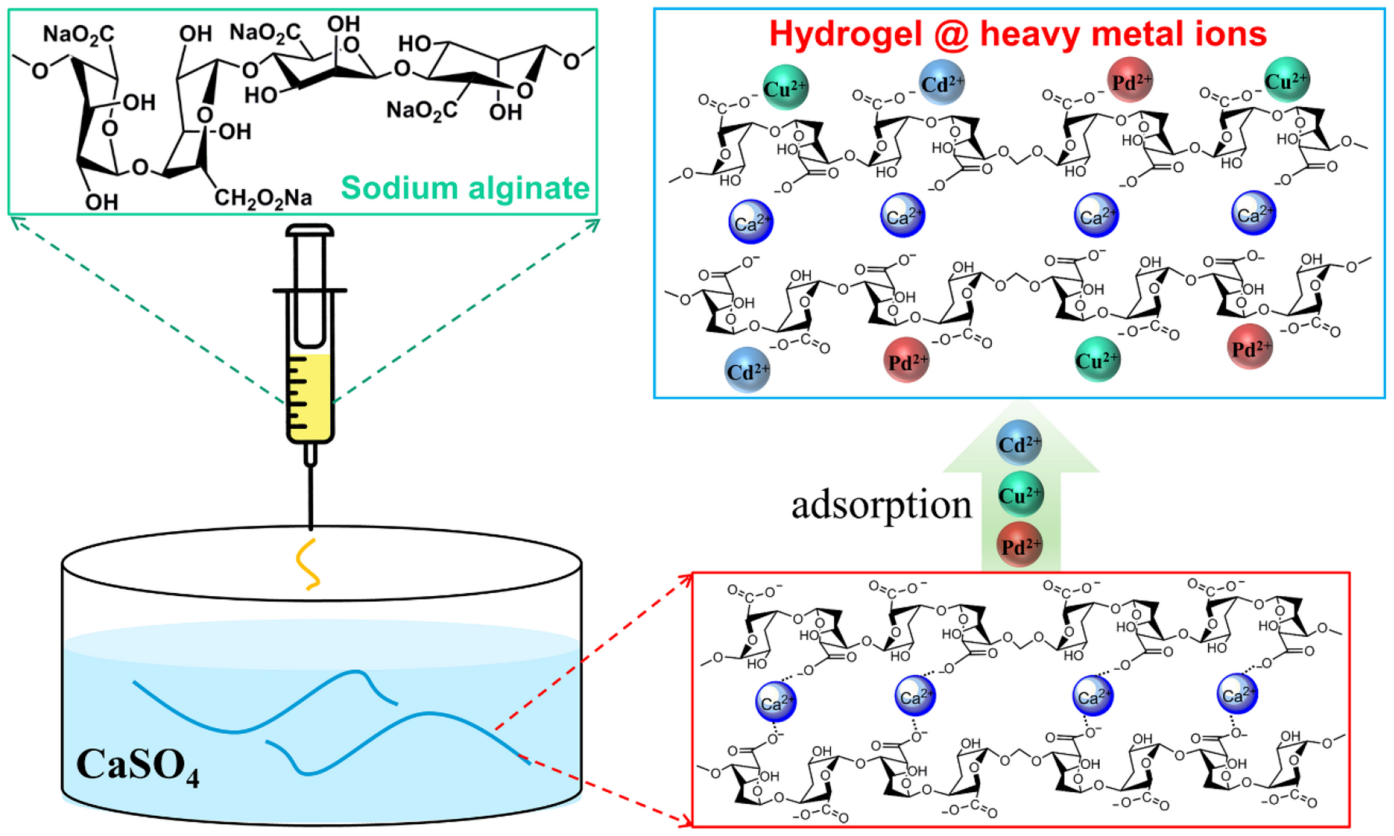
Schematic illustration of the AFH preparation process and their adsorption for heavy metal ions. SA solution is injected into a super-saturated solution of CaSO_4_, AFH is formed after crosslinking for 30 min. The adsorbent, AFH, is then added into an aqueous solution of heavy metal ions to remove toxic ions.

Natural sodium alginate (SA) was used as a macromolecule and Ca^2+^ served as crosslinker for fabricating AFH (Xie et al., 2012; Basu et al., 2017; Wang et al., 2019). Figure 1 shows a schematic illustration for the preparation of AFH and its adsorption process for heavy metal ions. With optimized concentrations of SA (52.7 mg·ml^−1^) and CaSO_4_ (2.5 mg·ml^−1^) and continuous injection of SA solution into the CaSO_4_ solution, fibrous-shaped hydrogels with a diameter of 1 mm were obtained after crosslinking for 30 min. The as-prepared hydrogel contains a lot of adsorption sites that can interact with heavy metal ions (Mahou et al., 2015).

**Figure 2:**
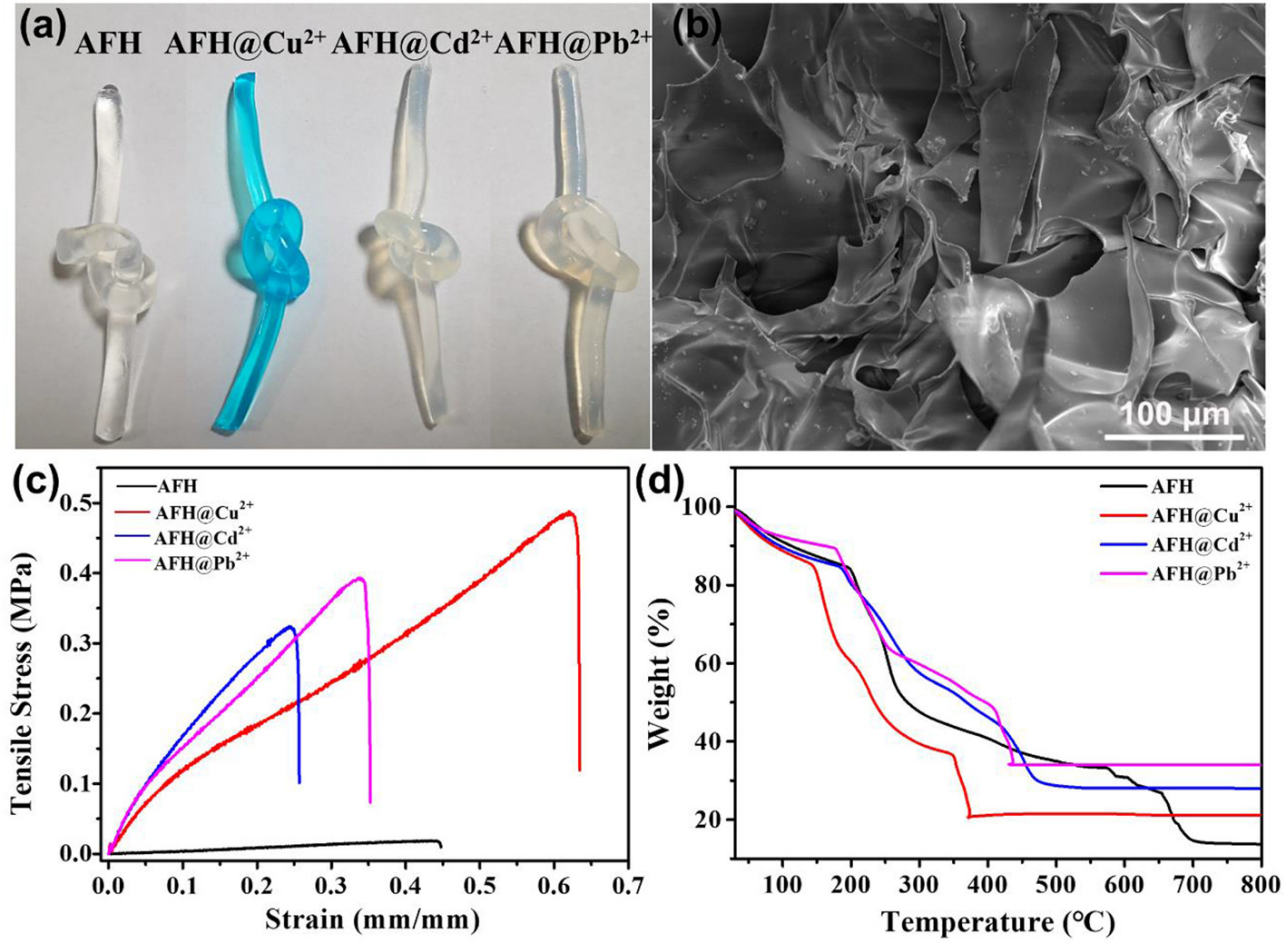
**(a)** Optical images of AFH without and with heavy metal ions adsorbed, **(b)** SEM image of AFH, **(c)** strain–stress curves of AFH with Cu^2+^/Cd^2+^/Pb^2+^ ion adsorbed, **(d)** TGA curve of without and with heavy metal ions adsorbed.

As shown in Figure 2a, the freshly prepared AFH are colorless and transparent, and the color turns blue after the adsorption of Cu^2+^ ions. However, AFH with Cd^2+^ and Pb^2+^ are light colored. SEM images in Figure 2b display that the AFH contain lots of voids, and these voids could result in more adsorption channels for heavy metal ions including Cu^2+^, Cd^2+^, and Pb^2+^. Both the heavy metal ion-adsorbed and non-adsorbed AFH have certain toughness and strength, and this is very convenient for recovery after adsorption of heavy metal ions. It is worth noting that AFH exhibits enhanced mechanical strength after the adsorption of heavy metal ions (Figure 2c). Thermal gravimetric analysis (TGA) can be used to analyze the weight loss of dried hydrogel material with temperature. As shown in Figure 2d, there are three stages during the thermal decomposition of blank AFH. The first stage (room temperature to about 190°C) can be attributed to dehydration, and the weight loss is about 12 wt.% (Shamshina et al., 2014). The second stage (~200 to 280°C) is due to the breakdown of alginate polymer chains and has a weight loss of about 30.1 wt.%. The third stage (654–700°C) is due to the evaporation of CO_2_ and formation of carbonized materials, and the weight loss is about 12.5 wt.% (Narayanan and Han, 2017; Dipankar et al., 2018). Due to the combination of adsorbed heavy metal ions and functional groups in the hydrogel, AFH with heavy metal ions adsorbed show less weight loss compared with blank AFH, and they also have lower stable temperature.

**Figure 3:**
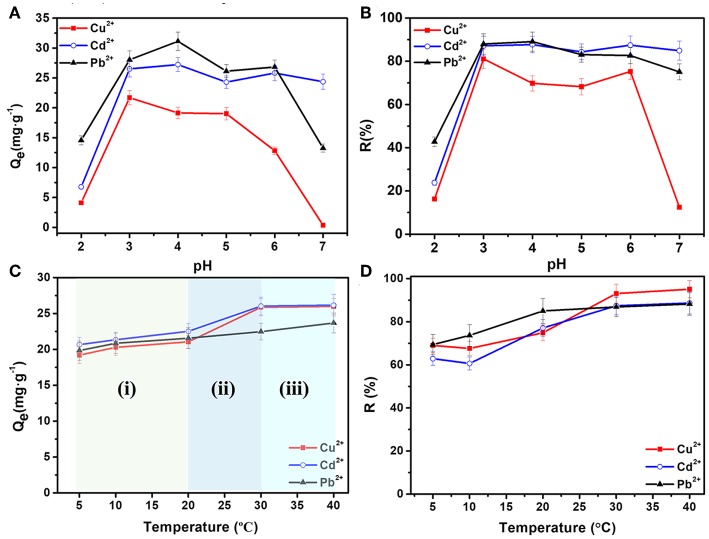
Effects of pH values on **(A)** equilibrium adsorption capacities and **(B)** removal ratios; effect of temperature on **(C)** equilibrium adsorption capacities and **(D)** removal ratios of Cd^2+^, Cu^2+^, and Pb^2+^ ions.

To study the adsorption performances, previously dried hydrogel samples were used to remove heavy metal ions. Figures 3A,B illustrate the effect of pH values on the adsorption capacity and removal ratios of Cd^2+^, Cu^2+^, and Pb^2+^ ions by AFH. *C*_0_ is 40 mg·L^−1^, *V*_0_ is 20 mL, *M* is 30 mg, *t* is 24 h, and *T* is 30°C. The *Q*_*e*_ values first increased and then decreased with an increase in pH values from 2.0 to 7.0. At lower pH, the concentration of hydrogen ions in SA chains is higher, and thus hydrogen ions compete more strongly for the electrons pair than the heavy metal ions. As a result, the removal of Cd^2+^, Cu^2+^, and Pb^2+^ ions is not effective. As the pH increases, protonation of the carboxyl group decreases gradually, more and more carboxyl groups can chelate with heavy metal ions resulting in enhanced adsorption (Wu and Li, 2013; Li et al., 2016; Wahab et al., 2019). However, the increasing concentration of OH^−^ as pH increases to 7.0 can causes some of the heavy metal ions to precipitate. Thus, removal of Cu^2+^ and Pb^2+^ ions is not satisfactory at pH of 7.0 (Panchan et al., 2018). The optimal pH values are 4.0 for Cd^2+^ and Pb^2+^ ions, and 3.0 for Cu^2+^ ions, respectively. Also, the corresponding removal ratios (*R%*) for each heavy metal ion is more than 80%.

Figures 3C,D illustrate the effect of temperature on the removing of Cd^2+^, Cu^2+^, and Pb^2+^ ions, in which *C*_0_ was 40 mg·L^−1^, *V*_0_ was 20 mL, *M* was 30 mg, *t* was 24 h, and pH was 4.0. The equilibrium adsorption capacities (*Q*_*e*_) can be analyzed in three stages. In the first stage (i) from 5 to 20°C, *Q*_*e*_ increases slightly with temperature. In the second stage (ii) from 20 to 30°C, the adsorption capacity has a significant augment. Whereas, in the third stage (iii) from 30 to 40°C, the adsorption capacities and removal ratios have only slight changes. In general, the adsorption capacities and removal ratios of heavy metal ions become a little larger at higher temperature. There are two possible reasons for this. One is that the higher degree of crosslinking at higher temperature can result in an increasing number of pores in AFH, which further increases the number of adsorption active sites. Another one can be attributed to the decrease in boundary layer thickness of the adsorbent, causing a lower mass transfer resistance of adsorbate (Kalagasidis Krušić et al., 2012).

**Figure 4:**
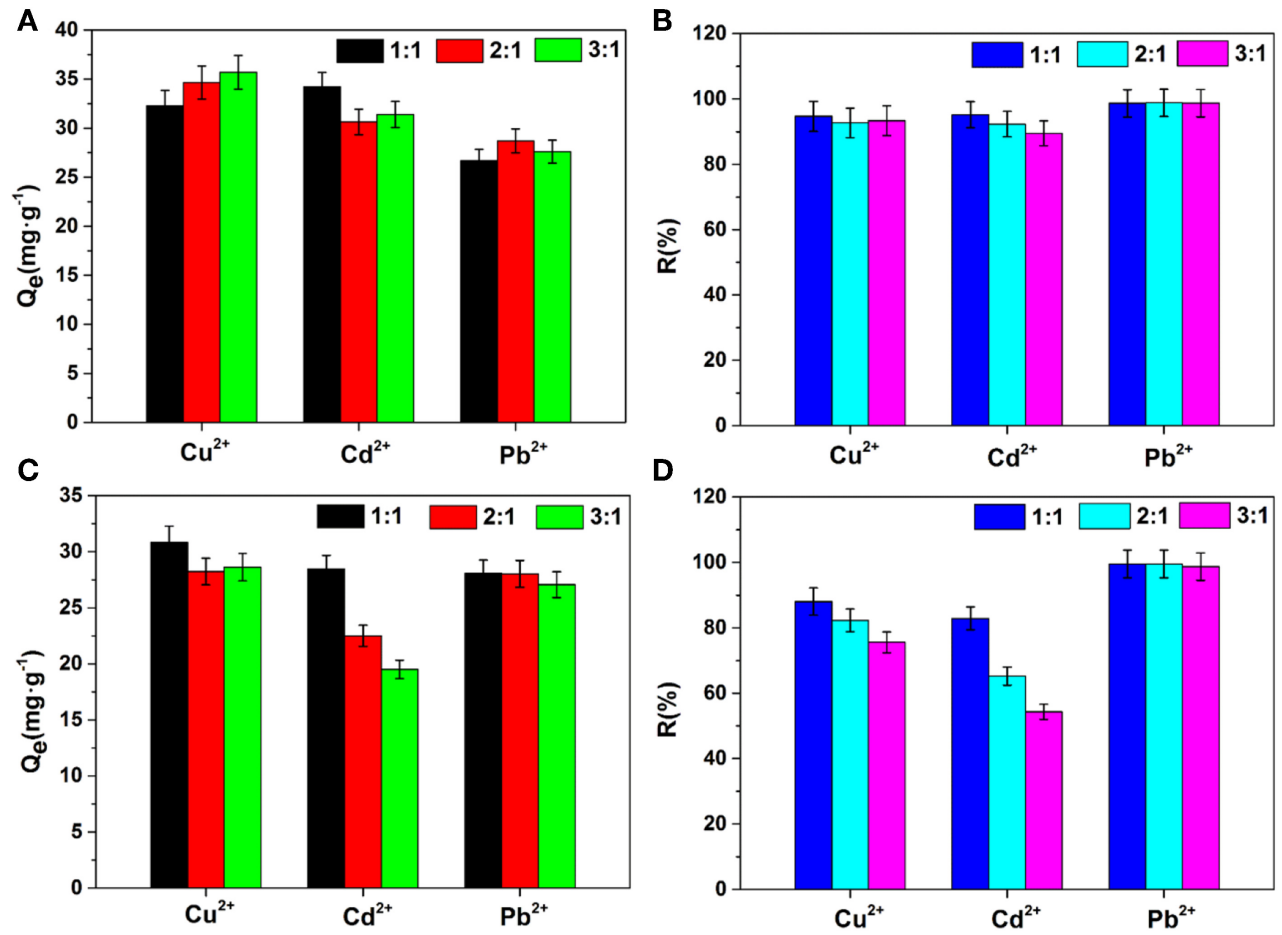
Effects of interfering ions on adsorption capacities and removal ratios **(A,B)** Na^+^ ion, **(C,D)** Ca^2+^ ion. Concentration ratios of interfering ions to heavy metal ions are 1:1, 2:1, and 3:1.

Many kinds of metal ions always coexist in the sewage. To better understand effects of other ions on the removal of heavy metal ions, Na^+^ and Ca^2+^ ions were selected as interfering ions for adsorption experiments, and the effects of the concentration ratios of interfering ions to heavy metal ions (1:1, 2:1, and 3:1) were also studied. As shown in Figure 4, the order of influence for the three heavy metal ions in this study is Cd^2+^ > Cu^2+^ > Pb^2+^. Na^+^ ions had only a slight effect on the adsorption capacities and removal ratios even when the concentration of Na^+^ was high. Among the three heavy metal ions, the adsorption for Pb^2+^ is almost unaffected by the presence of Na^+^ and Ca^2+^ ions, indicating its good anti-interfering absorbability of Pb^2+^. However, the removal ratio of Cd^2+^ decreased from 82.91 to 54.30% with an increasing concentration of Ca^2+^ ions. This is because Ca^2+^, as interfering ions in solution, may combine with adsorption sites inside AFH, and thus, Ca^2+^ has a competitive relationship with the adsorption of heavy metal ions. The ability of sodium alginate to bind with divalent cations is in the order of Pb^2+^ > Cu^2+^ > Cd^2+^, so Cd^2+^ is affected the most (Zhou et al., 2018). In addition, when interfering ions with high ionic strength coexist with heavy metal ions, the large concentration difference reduces the porosity and effective adsorption sites of hydrogel, and thus affect adsorption of heavy metal ions (Yang and Jiang, 2014).

**Figure 5:**
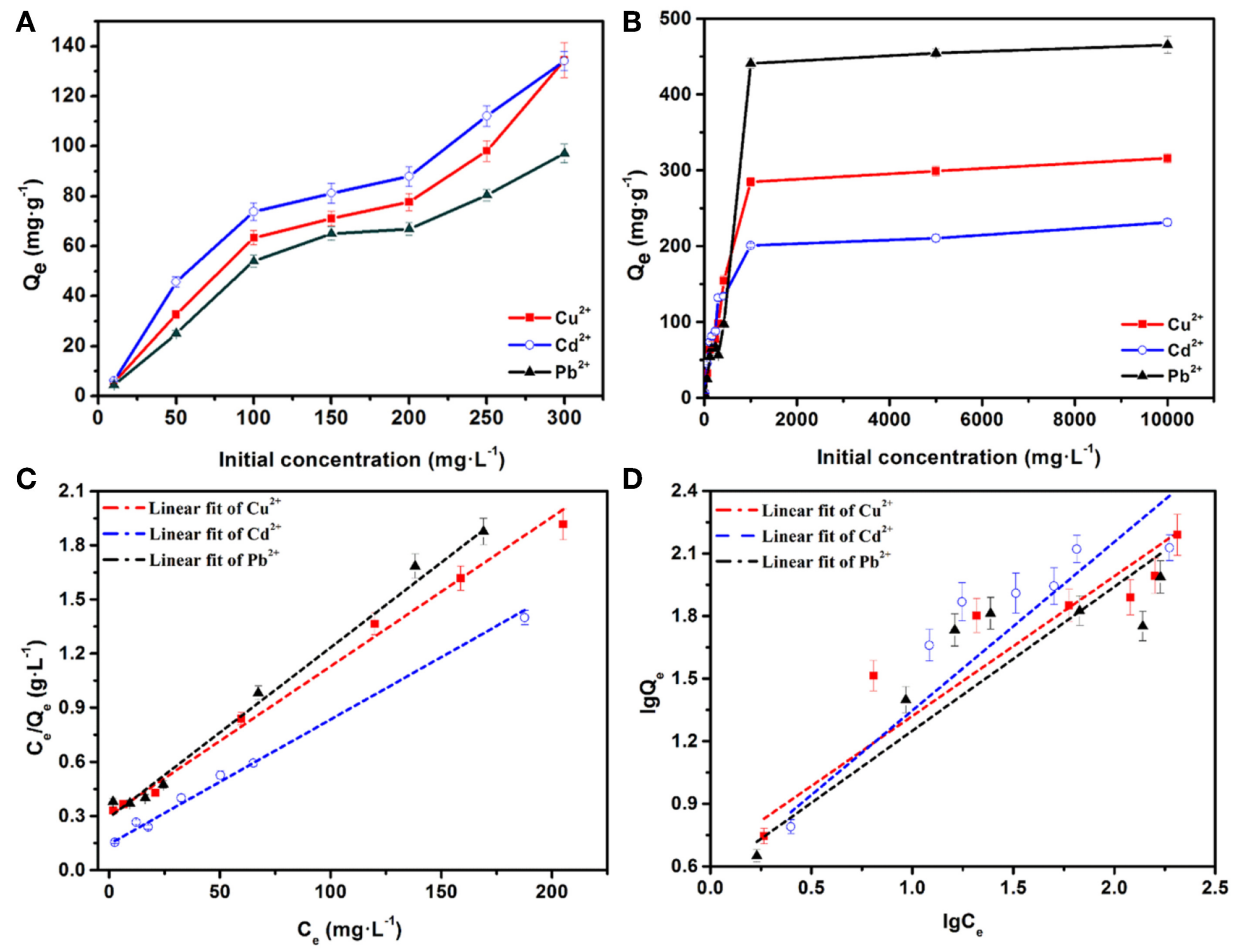
**(A,B)** Effects of initial concentrations on the adsorption capacities of Cd^2+^, Cu^2+^, and Pb^2+^ ions, the equilibrium isotherm analysis using the **(C)** Langmuir and **(D)** Freundlich models.

Figures 5A,B illustrate the effects of different initial concentrations of heavy metal ions on the adsorption by AFH. The experimental conditions were 24 h of adsorption at 303 K with 30 mg of AFH. The initial heavy metal ion concentrations were found to have a significant effect on the adsorption capacities. At relatively low ion concentrations (0–300 mg·L^−1^), the adsorption capacities increased very quickly with the increasing of initial ion concentrations. They then reach an equilibrium adsorption capacity when the concentrations are very high (1,000–10,000 mg·L^−1^). There are two explanations for this phenomenon. First, the quality of the hydrogel is constant throughout the adsorption process. When the ions concentration increases, the chance of collision between heavy metal ions and the hydrogel increases, and this results in an increase in equilibrium adsorption capacity. Second, the number of active adsorbed sites on AFH is definite, and it meets the adsorption requirement when the ion concentration is low. In contrast, the active sites gradually saturate as an increasing amount of heavy metal ions react with them, and this means that the adsorption can reach saturation (Zhang et al., 2018).

To better understand the adsorption isotherms, Langmuir and Freundlich models were applied to simulate experimental results. Figures 5C,D show the fitting curves for the adsorption of Cu^2+^, Cd^2+^, and Pb^2+^ ions using Langmuir and Freundlich models, respectively. As displayed in Table 1, the correlation coefficients (*R*^2^) calculated using Langmuir model are 0.993 (Cd^2+^), 0.974 (Cu^2+^), and 0.974 (Pb^2+^), which are higher than the *R*^2^ values obtained using the Freundlich model. Therefore, the adsorptions of Cd^2+^, Cu^2+^, and Pb^2+^ fit the Langmuir isotherm model better than the Freundlich isotherm model. In addition, the adsorption of each of these heavy metal ions is a monolayer adsorption process. The separation factors (*R*_*L*_) for Cu^2+^, Cd^2+^, and Pb^2+^ ions are 0.059–0.862, 0.032–0.769, and 0.031–0.763, respectively. All of the separation factors were between 0 and 1, indicating that the adsorption processes are very favorable (Yan et al., 2011). The saturated adsorption capacities calculated using the Langmuir isotherm model are 232.35 mg·g^−1^ (Cd^2+^), 315.92 mg·g^−1^ (Cu^2+^), and 465.22 mg·g^−1^ (Pb^2+^), and these are quite close to the experimental results (Figure 5B). As shown in Table S1, the saturated adsorption capacities of the as-prepared AFH were very competitive compared with other adsorbents.

**Table 1:** Langmuir and Freundlich isotherm parameters of the heavy metal ions adsorption.

**Metal ions**	**Langmuir isotherm**				**Freundlich isotherm**		
	***R*^**2**^**	***K_***L***_* (L·mg^**−1**^)**	***Q_***m***_*(mg·g^**−1**^)**	***R_***L***_***	***R*^**2**^**	***K_***F***_***(mg^1-1/n^·**L^**1/n**^ ·g^**−1**^)**	***n***
Cu^2+^	0.993	0.016	315.92	0.059–0.862	0.877	5.76	1.862
Cd^2+^	0.974	0.030	232.32	0.032–0.769	0.773	6.30	1.920
Pb^2+^	0.974	0.031	465.22	0.031–0.763	0.893	4.35	1.565

**Figure 6:**
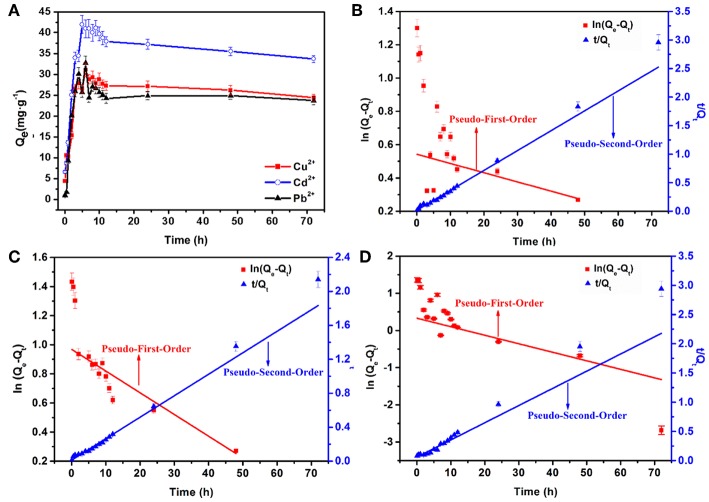
**(A)** Effects of adsorption time on the capacities of AFH for Cu^2+^, Cd^2+^, and Pb^2+^ ions, the plots of pseudo-first-order and pseudo-second-order kinetics models for **(B)** Cu^2+^, **(C)** Cd^2+^ and **(D)** Pb^2+^.

Adsorption kinetic is also one significant aspect of an adsorption system that must be studied including the mass transport and chemical reaction processes. Figure 6A illustrates the effects of reaction time on the adsorption capacities of Cd^2+^, Cu^2+^, and Pb^2+^ within 72 h at 303 K with an initial concentration of 70 mg L^−1^. Three stages are observed during the adsorption process. In the first adsorption stage (0–5 h), *Q*_*e*_ increases rapidly, *Q*_*e*_ then gradually decreases in the second stage (5–12 h), and in the third stage (12–72 h) reaches an equilibrium. The *Q*_*e*_ values for Cd^2+^, Cu^2+^, and Pb^2+^ ions at 12 h are 37.8, 27.2, and 24.2 mg·g^−1^, respectively, and the corresponding *R* values are 91.8, 87.4, and 81.1%, respectively (as shown in Figure S1). The pseudo-first-order and pseudo-second-order kinetics models were applied to explore the kinetics processes of heavy metal ions adsorption. The plots are shown in Figures 6B–D, and the calculated corresponding parameters are displayed in Table S2. From the fitting data using the pseudo-second-order kinetics equation, *R*^2^ is higher than that obtained by the pseudo-first-order model. Also, the theoretical equilibrium adsorption capacities (*Q*_*e*,2_) is quite close to the experimental results (*Q*_*e,exp*_). As mentioned, the results suggest that the main rate-limiting factor is chemisorption in the adsorption process (Yan et al., 2011; Li et al., 2016). The rate constants reflect the speed at which the heavy metal ions to reach adsorption equilibrium. As *K*_2_ increased, the time required to reach equilibrium is shorter. Therefore, the adsorption rate order of AFH for these three heavy metal ions is: Cu^2+^ > Cd^2+^ > Pb^2+^.

Many kinds of heavy metal ions always coexist in the waste water, and so it is essential to study the adsorption competitiveness among different metal ions. The experimental conditions were 24 h of adsorption at 303 K with an initial heavy metal ion solution concentration of 40 mg·L^−1^, and 30 mg of AFH. The partition coefficient (*K*_*d*_) and selectivity coefficient (α) were used to characterize the competitiveness of a certain heavy metal ion. Table S3 shows that Pb^2+^ has the strongest competitiveness when the three ions coexisted. The adsorption capacity between heavy metal ions and AFH is mainly related to the electronegativity and hydration radius of heavy metal ions. The electronegativities of Cu^2+^, Cd^2+^, and Pb^2+^ are 1.90, 1.69, and 2.33, respectively. The stronger electronegativity of a heavy metal ion induces the enhanced adsorption ability. Furthermore, the ion with the smaller hydration radii has stronger competitiveness, and Pb^2+^ ions has the smallest hydration radii among the three heavy metal ions (Cu^2+^: 4.19 Å, Cd^2+^: 4.26 Å, and Pb^2+^: 4.01 Å; Volkov et al., 1997).

## Conclusion

In summary, sodium alginate fibroid hydrogels are prepared through a Ca^2+^ ion-induced crosslinking method, and their application in the adsorption of heavy metals is investigated. AFH has a network structure and shows excellent potential in the treatment of wastewater including Cu^2+^, Cd^2+^, and Pb^2+^. And the enhanced mechanical strength after the adsorption of heavy metal ions will be very convenient for its recovery. The adsorption capacities are pH-sensitive, especially for Cu^2+^ and Pb^2+^. With an increase in temperature, the adsorption capacities and removal ratios are enhanced. The coexistence of Na^+^ barely affects the adsorption of heavy metal ions, but the coexistence of Ca^2+^ leads to the reduced removal of Cd^2+^. The adsorption process fits the Langmuir isotherm model and pseudo-second-order kinetic, and the main rate-limiting factor is chemisorption. Pb^2+^ has the strongest competitiveness when the three ions coexist. It is noteworthy that the saturated adsorption capacities of AFH for Cu^2+^, Cd^2+^, and Pb^2+^ are as high as 315.92, 232.35, and 465.22 mg·g^−1^, respectively. These values are comparable to that of reported absorbents, suggesting AFH is an effective adsorbent and has great potential applications in the treatment of heavy metal ions contaminated wastewater.

## Data Availability Statement

The datasets generated for this study are available on request to the corresponding author.

## Author Contributions

YC and ZY conceived this study. CK, XZ, and YL made the experiments and analyzed the data. CK made the draft of the manuscript with support of SY, YC, and ZY. All authors have made direct contribution to the work and approved this paper for publication.

### Conflict of Interest

The authors declare that the research was conducted in the absence of any commercial or financial relationships that could be construed as a potential conflict of interest.
