# Supplementary material for: Ion-Induced Synthesis of Alginate Fibroid Hydrogel for Heavy Metal Ions Removal
Source: Front Chem. 2020 Jan 10;7:905. doi: 10.3389/fchem.2019.00905 (PMC6966715; doi:10.3389/fchem.2019.00905)
Supplement: Supplementary file 1 [file Table_1.DOCX]

**Ion-induced synthesis of alginate fibroid hydrogel for heavy metal ions removal**

**Chuncai Kong^1^, Xueqi Zhao^1^, Yingju Li^1^, Sen Yang^1^, Yongmei Chen^2^*, Zhimao Yang^1^***

^1^School of Science, MOE Key Laboratory for Non-Equilibrium Synthesis and Modulation of Condensed Matter, State Key Laboratory for Mechanical Behavior of Materials and Xi’an Jiaotong University Suzhou Academy, Xi’an Jiaotong University, Xi’an 710049, Shaanxi, P. R. China

^2^College of Bioresources Chemical and Materials Engineering, Shaanxi University of Science & Technology, Key Laboratory of Leather Cleaner Production, China National Light Industry, Xi’an 710021, P. R. China

*** Correspondence:**Corresponding Author
chenym@mail.xjtu.edu.cn (Y.M. Chen)

zmyang@mail.xjtu.edu.cn (Z.M. Yang)


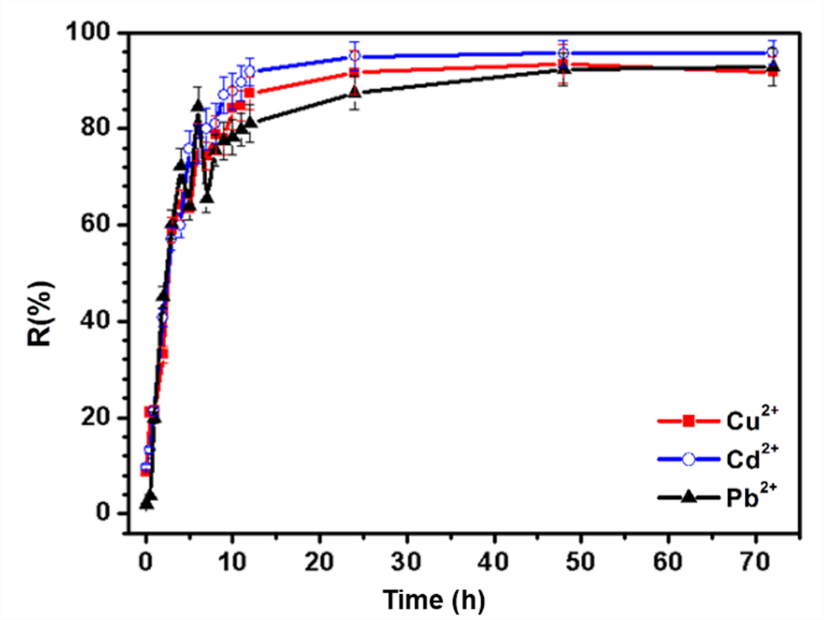


FIGURE S1 Effect of time on the removal ratios of SAFH for Cu^2+^, Cd^2+^ and Pb^2+^.

TABLE S1 Comparison of saturated adsorption capacities (mg·g^-1^) of different materials

| Adsorbents | Cu^2+^ | Cd^2+^ | Pb^2+^ | Reference |
| --- | --- | --- | --- | --- |
| SAFH | 315.92 | 232.35 | 465.22 | This work |
| PVA/PAA gel | — | 115.88 | 194.99 | (Chu et al. 2015) |
| PSA-GO gel | — | 238.30 | — | (Xu et al. 2015) |
| biochar | 34.20 | 28.10 | 153.10 | (Wang et al. 2015) |
| AMPS | 100.86 | 134.66 | 120.14 | (Ozay et al. 2009) |
| Fraxinus excelsior tree leaves | 33.10 | 67.20 | 172.00 | (Sangi et al. 2008) |
| PAM/TGA/DHBA | — | 294.08 | 452.25 | (Mohammadi et al. 2017) |
| PAAm/TGA |  | 291.73 | 345.00 | (Mohammadi, Shangbin, Berkland,Liang 2017) |
| GO-DPA | 347.00 | 253.00 | 360.00 | (Zare-Dorabei et al. 2016) |

TABLE S2 Kinetic rate constants and calculated adsorption capacity of heavy metal ions

| Metal ions | *Q_e,exp_* (mg·g^-1^) | Pseudo-first-order model | | | Pseudo-second-order model | | |
| --- | --- | --- | --- | --- | --- | --- | --- |
|  |  | *R^2^* | *Q_e,1_* (mg·g^-1^) | *K_1_* (min^-1^) | *R^2^* | *Q_e,2_* (mg·g^-1^) | *K_2_* (g·(mg•min)^-1^) |
| Cu^2+^ | 24.34 | 0.156 | 3.48 | 0.013 | 0.956 | 28.72 | 0.065 |
| Cd^2+^ | 33.64 | 0.860 | 9.29 | 0.034 | 0.937 | 39.56 | 0.051 |
| Pb^2+^ | 23.66 | 0.232 | 2.14 | 0.053 | 0.912 | 34.00 | 0.014 |

TABLE S3 Partition and selectivity coefficients of competitive adsorption on heavy metal ions

| Metal ions | *K_d_* | | | *α* | | |
| --- | --- | --- | --- | --- | --- | --- |
|  | Cu^2+^ | Cd^2+^ | Pb^2+^ | Pb^2+^/ Cu^2+^ | Pb^2+^/ Cd^2+^ | Cu^2+^/ Cd^2+^ |
| Cu^2+^ + Cd^2+^ | 0.509 | 0.491 | — | — | — | 1.036 |
| Cu^2+^ + Pb^2+^ | 0.494 | — | 0.506 | 1.025 | — | — |
| Cd^2+^ + Pb^2+^ | — | 0.493 | 0.507 | — | 1.028 | — |
| Cu^2+^ + Cd^2+^ + Pb^2+^ | 0.331 | 0.312 | 0.357 | 1.079 | 1.144 | 1.059 |

**REFERENCES**

Chu L, Liu C, Zhou G, Xu R, Tang Y, Zeng Z, Luo S (2015) A double network gel as low cost and easy recycle adsorbent: Highly efficient removal of Cd(II) and Pb(II) pollutants from wastewater J Hazard Mater 300:153-160 https://doi.org/10.1016/j.jhazmat.2015.06.070

Li J, Tong J, Li X, Yang Z, Zhang Y, Diao G (2016) Facile microfluidic synthesis of copolymer hydrogel beads for the removal of heavy metal ions J Mater Sci 51:10375-10385 10.1007/s10853-016-0258-0

Meena AK, Mishra GK, Rai PK, Rajagopal C, Nagar PN (2005) Removal of heavy metal ions from aqueous solutions using carbon aerogel as an adsorbent J Hazard Mater 122:161-170 https://doi.org/10.1016/j.jhazmat.2005.03.024

Mohammadi Z, Shangbin S, Berkland C, Liang J-t (2017) Chelator-mimetic multi-functionalized hydrogel: Highly efficient and reusable sorbent for Cd, Pb, and As removal from waste water Chem Eng J 307:496-502 https://doi.org/10.1016/j.cej.2016.08.121

Ozay O, Ekici S, Baran Y, Aktas N, Sahiner N (2009) Removal of toxic metal ions with magnetic hydrogels Water Res 43:4403-4411 https://doi.org/10.1016/j.watres.2009.06.058

Sangi MR, Shahmoradi A, Zolgharnein J, Azimi GH, Ghorbandoost M (2008) Removal and recovery of heavy metals from aqueous solution using Ulmus carpinifolia and Fraxinus excelsior tree leaves J Hazard Mater 155:513-522 https://doi.org/10.1016/j.jhazmat.2007.11.110

Wang H, Gao B, Wang S, Fang J, Xue Y, Yang K (2015) Removal of Pb(II), Cu(II), and Cd(II) from aqueous solutions by biochar derived from KMnO4 treated hickory wood Bioresource Technol 197:356-362 https://doi.org/10.1016/j.biortech.2015.08.132

Xu R, Zhou G, Tang Y, Chu L, Liu C, Zeng Z, Luo S (2015) New double network hydrogel adsorbent: Highly efficient removal of Cd(II) and Mn(II) ions in aqueous solution Chem Eng J 275:179-188 https://doi.org/10.1016/j.cej.2015.04.040

Yan H, Dai J, Yang Z, Yang H, Cheng R (2011) Enhanced and selective adsorption of copper(II) ions on surface carboxymethylated chitosan hydrogel beads Chem Eng J 174:586-594 https://doi.org/10.1016/j.cej.2011.09.064

Zare-Dorabei R, Ferdowsi SM, Barzin A, Tadjarodi A (2016) Highly efficient simultaneous ultrasonic-assisted adsorption of Pb(II), Cd(II), Ni(II) and Cu (II) ions from aqueous solutions by graphene oxide modified with 2,2′-dipyridylamine: Central composite design optimization Ultrason Sonochem 32:265-276 https://doi.org/10.1016/j.ultsonch.2016.03.020

Zhang M, Song L, Jiang H, Li S, Shao Y, Yang J, Li J (2017) Biomass based hydrogel as an adsorbent for the fast removal of heavy metal ions from aqueous solutions J Mater Chem 5:3434-3446 10.1039/C6TA10513K

Zhang W et al. (2018) A facile synthesis of core-shell/bead-like poly (vinyl alcohol)/alginate@PAM with good adsorption capacity, high adaptability and stability towards Cu(Ⅱ) removal Chem Eng J 351:462-472 https://doi.org/10.1016/j.cej.2018.06.129
